# Aberrant Glycosylation as Biomarker for Cancer: Focus on CD43

**DOI:** 10.1155/2014/742831

**Published:** 2014-02-13

**Authors:** Franca Maria Tuccillo, Annamaria de Laurentiis, Camillo Palmieri, Giuseppe Fiume, Patrizia Bonelli, Antonella Borrelli, Pierfrancesco Tassone, Iris Scala, Franco Maria Buonaguro, Ileana Quinto, Giuseppe Scala

**Affiliations:** ^1^Molecular Biology and Viral Oncogenesis Unit, Department of Experimental Oncology, Istituto Nazionale Tumori “Fondazione G. Pascale”—IRCCS, Naples, Italy; ^2^Department of Experimental and Clinical Medicine, University “Magna Graecia” of Catanzaro, Catanzaro, Italy; ^3^Department of Pediatrics, University of Naples “Federico II”, Naples, Italy

## Abstract

Glycosylation is a posttranslational modification of proteins playing a major role in cell signalling, immune recognition, and cell-cell interaction because of their glycan branches conferring structure variability and binding specificity to lectin ligands. Aberrant expression of glycan structures as well as occurrence of truncated structures, precursors, or novel structures of glycan may affect ligand-receptor interactions and thus interfere with regulation of cell adhesion, migration, and proliferation. Indeed, aberrant glycosylation represents a hallmark of cancer, reflecting cancer-specific changes in glycan biosynthesis pathways such as the altered expression of glycosyltransferases and glycosidases. Most studies have been carried out to identify changes in serum glycan structures. In most cancers, fucosylation and sialylation are significantly modified. Thus, aberrations in glycan structures can be used as targets to improve existing serum cancer biomarkers. The ability to distinguish differences in the glycosylation of proteins between cancer and control patients emphasizes glycobiology as a promising field for potential biomarker identification. In this review, we discuss the aberrant protein glycosylation associated with human cancer and the identification of protein glycoforms as cancer biomarkers. In particular, we will focus on the aberrant CD43 glycosylation as cancer biomarker and the potential to exploit the UN1 monoclonal antibody (UN1 mAb) to identify aberrant CD43 glycoforms.

## 1. Introduction

**Figure 1:**
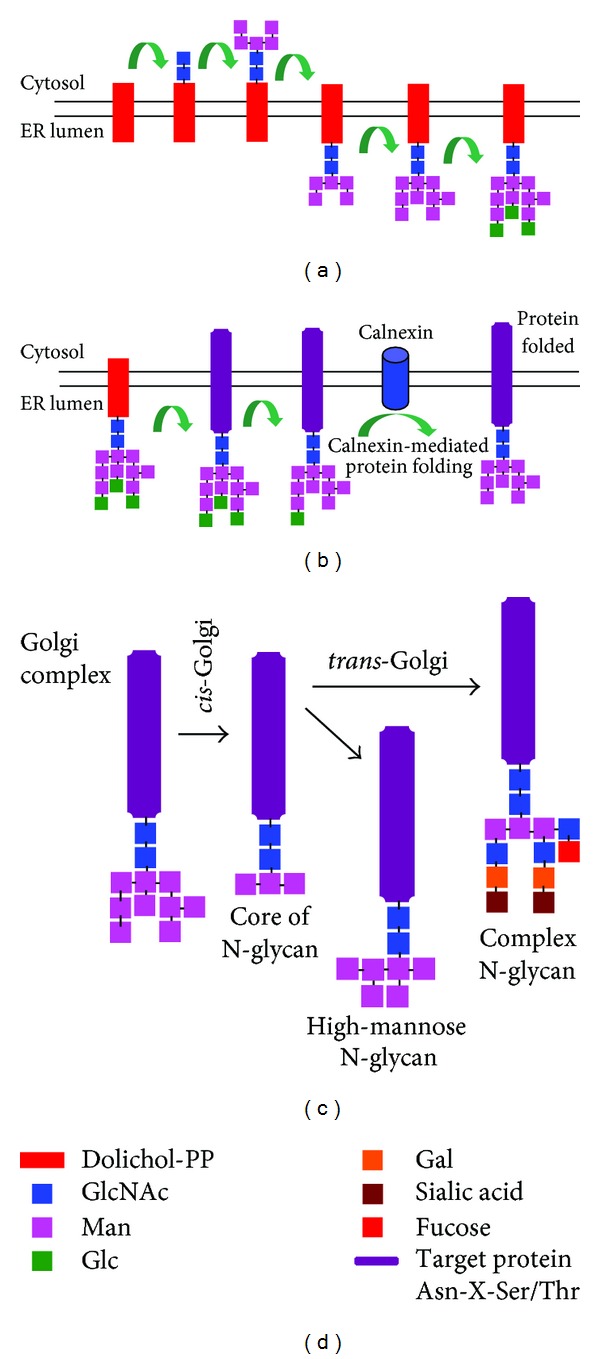
Schematic representation of N-glycosylation process. N-Glycosylation is an evolutionary conserved stepwise process that can be summarized as follows. (a) The assembling of the precursor oligosaccharide occurs at the cytoplasmic side of endoplasmic reticulum (ER) and starts with dolichol-pyrophosphate (Dol-PP), which is a nucleotide linked to a monosaccharide on a lipid carrier, an isoprenoid compound (90–100 carbon atoms total). The sequential incorporation of monosaccharides N-acetyl-glucosamine (GlcNAc) and Mannose (Man) is catalyzed by various glycosyltransferases. Once the intermediate Dol-PP-GlcNA_2_cMan_5_ is made, it flips to the luminal side of the ER, where four further residues of mannose and three residues of glucose (Glc) are added, leading to Dol-PP-GlcNAc_2_Man_9_Glc_3_. (b) When the proteins containing the consensus sequence for N-glycosylation (Asn-X-Ser and Asn-X-Thr) translocate to the ER, they are glycosylated by the oligosaccharyltransferase (OST) that catalyzes the transfer of the N-glycan to specific asparagine residues included within the consensus sequence (Asn-X-Ser/Thr) of the target proteins. After the oligosaccharide is transferred to the target protein, specific enzymes remove the three Glucose residues and one particular Mannose. The ER lumen contains a specific glycosyltransferase that binds to target protein, catalyzing the addition of Glucose residues only when the target protein is unfolded or misfolded. The newly glycosylated Glc_1_GlcNAc_2_Man_7−9_ oligosaccharides are then bound by two specific lectins, the ER-membrane-attached Calnexin or ER-luminal Calreticulin, which will allow the glycoprotein folding. Once folding is completed, the glucose residue is removed. (c) N-Glycosylated proteins move from ER to Golgi, where specific enzymes catalyse their sequential modifications to the Man_8_(GlcNAc)_2_ chains. In particular, in the cis-Golgi compartment, most Mannose residues of the original backbone chain are removed, leading to a N-glycan core structure constituted by GlcNAc_2_Man_3_. In mammals, two main groups of oligosaccharides linked to proteins are found: complex N-glycans and high-Mannose N-glycans. These structures are generated during the passage of the proteins toward the *trans*-side by the subsequent addition of Mannose residues (for the high-mannose N-glycans) and by addition of three residues of N-acetyl glucosamine (GlcNAc), two residues of Gal (galactose), two residues of NeuAc (N-acetylneuraminic acid) or sialic acid, and a single fucose residue (for the complex N-glycans). (d) Graphic legend of described structures.

**Figure 2:**
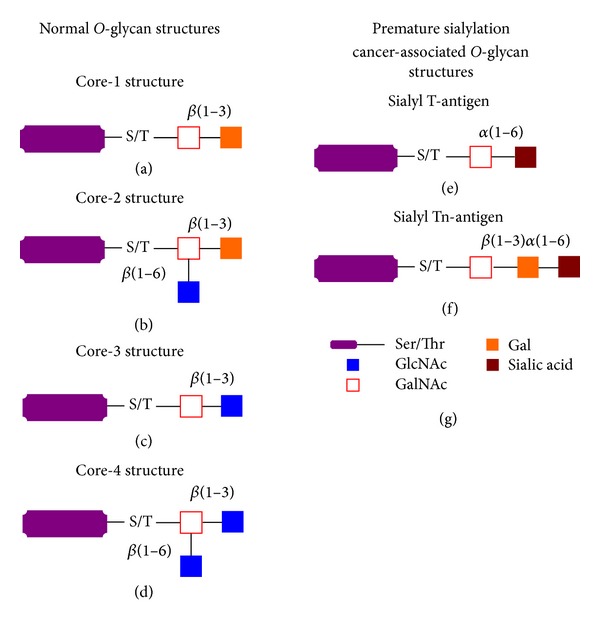
Mucin-type *O*-glycosylation. Different forms of *O*-glycosylation of proteins occur in animals. Mucin-type *O*-glycosylation is the most abundant form of protein *O*-glycosylation and consists of glycans attached via *O*-linked N-acetylgalactosamine (GalNAc) to serine and threonine residues. Other types of *O*-glycosylation include mannose-, galactose- (added to a residue of hydroxylysine), fucose-, glucose-, xylose-, and N-acetylglucosamine-*O*-glycosylation. GalNAc-*O*-glycosylation originates in the Golgi apparatus after protein folding, while other types of *O*-glycosylation of proteins in the secretory pathway initiate in the ER. Mucin-type *O*-glycosylation is initiated by a large family of homologous proteins, named N-acetylgalactosamine (GalNAc)-transferase that catalyses the transfer of GalNAc from UDP-GalNAc to Ser or Thr residues of target glycoprotein. These enzymes are sequentially and functionally conserved across species and their expression is time and tissue specific, suggesting a very complex regulation. Up to 20 different isoforms of polypeptide N-acetyl-*α*-D-galactosaminyltransferases are known and many are specific for the sites of attachment of the GalNAc to serine/threonine residues, influencing the density and the specific position of the *O*-glycosylation of target proteins. Thereafter, specific glycosyltransferases can catalyse the addition to GalNAc of specific monosaccharides generating four common subtypes (Core-1-, Core-2-, Core-3-, and Core-4-*O*-glycan structures) based on differential monosaccharide linkage reactions to the GalNAc (GalNAc*α*-Ser/Thr). Most *O*-glycans contain the Core-1 subtype (a), which is generated by the addition of galactose to the GalNAc through a *β*1–3 linkage by the Core-1-(*β*1–3) galactosyltransferase. This structure is usually further extended by the addition of monosaccharides such as N-acetylglucosamine, galactose, N-acetylneuraminic acid, and fucose. (b) Core-2-*O*-glycans are generated by the addition of GlcNAc to the GalNAc through a *β*1–6 linkage. In order to generate Core-2-*O*-glycans, Core-1 structure is required as a substrate; thus, the Core-2 structure includes the Core-1 structure. The Core-2-*O*-glycan can be further extended into either a mono- or biantennary form by addition of multiple galactose (Gal(*β*1–4)GlcNAc) units and terminal linkages of fucose and sialic acid. (c) Core-3 subtype is generated by the addition of GlcNAc in a *β*1–3 linkage to the GalNAc, and it can be extended by the addition of GlcNAc in a *β*1–6 linkage, generating the Core-4-*O*-glycan (d), which also can be extended by addition of monosaccharides, such as galactose, fucose, and sialic acid, which results in the synthesis of a wide spectrum of *O*-glycan structures. In some cases, the biosynthesis of *O*-glycans is stopped by the addition of sialic acid residues in early biosynthesis leading to “dead ends” of *O*-glycan structures that cannot be further modified ((e), (f)). Truncated *O*-glycan structures are frequently found in cancer cells as tumour antigens, suggesting that aberrant glycosylation may contribute to cancer progression by modifying cell signalling, adhesion, and antigenicity. (g) Graphic legend of described structures.

Protein glycosylation is the most common and complex posttranslational modification involved in many physiological events, including protein folding and trafficking, cell-cell and cell-matrix interactions, cellular differentiations and the immune response [1–5]. Approximately, 1% of human genes are required for this specific process [6] with more than 50% of proteins being glycosylated according to SwissProt database [7]. In humans, protein-linked glycans can be divided into two main types: N-linked (linkage to the amide group of asparagine residues in the consensus sequence Asn-X-Ser/Thr) (Figure 1) and *O*-linked (linkage to the hydroxyl group of serine, threonine, or hydroxylysine) (Figures 2(a)–2(d)). Carbohydrate additions to proteins occur during or after translation as the nascent proteins transit through the endoplasmic reticulum (ER) and subsequent organelles in the secretory pathway of the cell. Glycosylation is not a template-based process such as DNA, RNA, or protein synthesis but is rather based on the balance achieved by the expression and activity levels of the different enzymes involved in the glycosylation process, such as glycosyltransferases and glycosidases, and on the availability of precursor monosaccharide molecules, which in turn depends on the availability of nutrient resources and expression of enzymes responsible for their synthesis and interconversion [3, 4, 7, 8]. This highly increases the complexity of the protein glycosylation process and the molecular microheterogeneity of glycoproteins [9]. Glycans exist as membrane-bound glycoconjugates or as secreted molecules, which can become integral parts of the extracellular matrix. In these locations, glycans can mediate cell adhesion and motility as well as intracellular signalling events [10]. Moreover, changes in glycan structures are associated with many physiological and pathological events such as cell growth, migration, and differentiation. Consequently, aberrant glycosylation occurring in cancer cells may influence cell proliferation, adhesion, and motility, as well as angiogenesis and metastasis [11, 12]. Neoplastic transformation is frequently associated with the expression of oncofetal antigens, which are usually expressed in fetal tissues and cancer tissues and not in normal adult tissues. These antigens are primarily glycoproteins and their glycan epitopes were identified by the first “tumour-specific” monoclonal antibodies [12]. Interest in these changes increased when a significant correlation between certain types of altered glycosylation and prognosis was observed. Glycan alterations in tumours, such as underexpression, truncation, or modified branching patterns, may correlate with cell growth and enhanced proliferative capacity of tumour cells. Of interest, despite the wide diversity of glycan structures, only a few distinct glycan changes are associated with malignant transformation and tumour progression. This suggests that specific glycosylation patterns correlate with tumour progression, likely due to gain of function in cell fitness and survival [12]. Altered patterns of protein glycosylation in transformed cells were initially exploited using plant lectins based on their specific binding to distinct sugar sequences [13]. In animals, lectins are involved in different biological functions, such as quality control of secreted proteins, cell-cell recognition, cell adhesion and motility, and pathogen-host recognition. Several lectins are found on the surface of immune cells and endothelial cells of blood vessels and act as extracellular matrix proteins and soluble adhesion molecules including mannose-binding proteins or galactose-binding lectins (galectins). The interactions of lectins with tumour cell glycans can promote tumour progression.

## 2. Altered Glycosylation Patterns in Cancer

Aberrant protein glycosylation is a well-established event in oncogenesis (Table 1) and cancer progression [40–42]. Mucin-type *O*-glycosylation, consisting of glycans attachment via *O*-linked N-acetylgalactosamine (GalNAc) to serine and threonine residues, is one of the most abundant forms of protein glycosylation in animals. Mucins (MUC) are large glycoproteins with a “rod-like” conformation that harbour many clustered glycosylated serines and threonines in tandem repeat structures (PTS domain). Fifty to ninety percent of the mucin molecule by weight consists of *O*-linked oligosaccharides (*O*-glycans). The *O*-glycan cores are usually lengthened and modified by addition of diverse sugars and sulphate esters, thus generating different structural conformations. Human MUC family includes 21 different members named from MUC1 to MUC21. Members of the mucin family are classified as secreted mucins (when released in the intercellular space) and transmembrane mucins (when expressed on the cellular surface). Secreted mucins constitute a protective barrier over the epithelium. *O*-Glycans of cell surface-bound mucins modulate the interactions with the environment through binding to lectins and antigens. Depending on the structures of *O*-glycan chains, mucins can have pro- or antiadhesive properties. Transmembrane mucins consist of a glycosylated extracellular domain, a hydrophobic transmembrane region, and a cytoplasmic tail. They contribute to the physical barrier and interact with cytoskeletal adaptor proteins and signalling molecules, thus contributing to cell adhesion and signalling pathways. Overexpression of mucins in carcinomas has been described for many years [31, 43–46]. In the normal polarized epithelium, mucins are expressed exclusively on the apical border of the cell sheets, toward the lumen of a hollow organ, and soluble mucins are secreted exclusively into the lumen. Malignant epithelial cells lose the correct polarity of mucins as they are expressed on the entire cellular membrane and secreted mucins can enter the extracellular space and body fluids, such as the blood [12, 44]. Mucins expressed in carcinoma cells from epithelia and in malignant haematopoietic cells are the ones exhibiting the most altered glycosylation pattern. Aberrant glycan structures can modify antigenic and adhesive properties of cancer cells and their potential to migrate. In addition to interfering with the specific binding to endogenous lectins, the elongated carbohydrate structures and the negative charge of mucins can sterically hinder the interaction of other adhesion molecules, such as cadherins and integrins, and their natural ligands, thus facilitating the detachment of tumour cells from the primary tumour during the process of metastasis. Moreover, mucins can physically hamper the interactions with the host immune cells, such as natural killer cells, and mask the presentation of antigenic peptides by the major histocompatibility complex [47].

Incomplete glycosylation is another abnormal feature of mucins found in human cancer. The expression of truncated *O*-glycans Tn, sialyl-Tn, and T antigens was reported in different human tumours [14–19, 21, 48] (Figures 2(e) and 2(f)). Since these structures are not expressed in normal tissues, they can cause immune responses in the patients. In fact, the expression of the T (Gal*β*1–3GalNAc-*α*1-*O*-Ser/Thr) and Tn (GalNAc-*α*1-*O*-Ser/Thr) antigens in carcinomas correlated with the presence of antibodies directed against these antigens and the prognosis of the disease [12, 15, 17]. Further, incomplete glycosylation leads to the expression of “naked” mucin polypeptides [44–46]. The aberrant expression of truncated *O*-glycans on cancer cells can be due to defects in the secretory pathway organelles (ER and Golgi) and to altered glycosyltransferase expression. These alterations can also depend on mutations in cosmc (Core-1 *β*3-Gal-T-specific molecular chaperone), a chaperone required for glycosyltransferase function [17, 20]. Expression of truncated *O*-glycans may also be due to the absence of glycosyltransferases responsible for the synthesis of core structures used as substrates for chain elongation [22], or overexpression of sialyltransferases responsible for the synthesis of sialyl-Tn and sialyl-T antigens [17, 49, 50].

Deregulated N-glycosylation of proteins has been observed in different pathologies and most biomarkers used for diagnosis, prognosis, and prediction of many cancers are N-linked glycosylated proteins [51, 52]. Several N-glycoproteins differentially expressed in some cancerous diseases have been reported, including the following: (1) upregulation of alpha-1-antichymotrypsin in both non-small-cell lung carcinoma (NSCLC) and hepatocellular carcinoma (HCC) [53, 54], (2) upregulation of galectin-3-binding protein (Gal3BP or Mac-2 BP) in both HCC and ovarian cancer [55, 56], (3) downregulation of insulin-like growth factor binding protein 3 in HCC and NSCLC [54, 56], and (4) increased expression of periostin in aggressive prostate cancer [57] and most ovarian cancer subtypes [55]. Altered branching of N-glycans is also involved in many neoplastic diseases. As the branching of N-glycans is involved in various biological functions, such as signal transduction, cell adhesion, cell motility, and cell proliferation, it is reasonable that the altered activity of distinct glycosyltransferases may be involved in various cancerous processes, including cancer cell migration, invasion, and metastasis [58]. In particular, some glycosyltransferases, including N-acetylglucosaminyltransferase III (GnT-III), N-acetylglucosaminyltransferase V (GnT-V), and *α*-1, 6-fucosyltransferase (Fut8), are strongly associated with many types of cancer. GnT-III catalyses the addition of GlcNAc to mannose linked to an underlying N-acetylglucosamine through a *β*-1, 4 linking, producing a “bisecting” GlcNAc linkage. The introduction of bisecting GlcNAc catalysed by GnT-III suppresses additional processing and elongation of N-glycans that are catalysed by other enzymes, such as GnT-IV, GnT-V, and GnT-VI [59]. GnT-III suppressed the lung metastatic foci in mice injected with GnT-III-transfected melanoma B16 cells as compared to mice treated with mock-transfected cells [60]; the mechanism of suppression of metastatic foci has been referred to the branching modifications of essential glycoproteins, such as epithelial growth factor receptor (EGFR), and adhesion molecules including integrin and cadherin [61–63]. On the contrary, GnT-V, which catalyses the formation of *β*-1, 6-GlcNAc branching structures, plays pivotal roles in the development of tumour cell invasiveness and metastasis. In particular, the increase in *β*-1, 6 branching of N-glycans depending on upregulated GnT-V activity was found to be associated with cancer growth and metastasis [32, 33, 64, 65]. Fut8 catalyses the transfer of the fucose residue from fucose-GDP to the innermost GlcNAc residue of hybrid and complex N-linked oligosaccharides in the specific position 6 of glycoproteins, resulting in core fucosylation through an *α*-1, 6 linking. Core-fucosylated glycoproteins are widely distributed in mammalian tissues and may be altered under pathological conditions, such as hepatocellular carcinoma and liver cirrhosis [34, 35]. Notably, the increase in Fut8 expression and activity was observed in thyroid papillary carcinomas and correlated to tumour size and lymph node metastasis, and thus Fut8 expression represents a biomarker of progression of thyroid papillary carcinomas [36].

## 3. Glycan-Based Serological Assays in Cancer

Aberrant glycosylation of membrane-bound or secreted glycans associated with neoplastic transformation has become cancer biomarkers. Serological assays are currently used in clinical practice to detect and quantify glycans in the serum of cancer patients. The measurement of circulating glycoconjugates is used for (a) diagnosis, (b) monitoring of clinical course under therapy, (c) detection of early disease recurrence, and (d) prognosis. Serum levels of glycoconjugates, such as SLe^a^ (CA19-9), STn (CA72-4), and the mucin glycoproteins MUC1 (CA15-3) and MUC16 (CA125) [66–69], are usually measured. These biomarkers cannot be used for cancer screening as they are broadly expressed by various types of cancer [70–72] and can also be produced in some nonneoplastic and inflammatory diseases [73], thus reducing the specificity required for screening [74]. Nevertheless CA125, for instance, remains the best available biomarker of ovarian cancer. CA125 is a transmembrane mucin that is released into the extracellular space by enzymatic cleavage. Elevated levels of CA125 are found in the serum of 50% of patients with stage I ovarian cancer and in about 25% of serum samples collected within 60 months preceding diagnosis of ovarian cancer [75]. Moreover, high serum levels of CA125 are detected in 80% of patients with epithelial ovarian cancer [67, 76]. In addition, the CA125 levels are directly correlated with the progression or regression of the disease, and the preoperative levels of CA125 are used to assess the prognosis of ovarian cancer patients. On the whole, CA125 is a valuable serum tumour marker for monitoring response to chemotherapy, detecting disease recurrence, and evaluating prognosis [77–79].

MUC1 is a transmembrane mucin overexpressed and aberrantly glycosylated in more than 90% of human breast cancer. As a consequence of neoplastic transformation and loss of epithelial cell polarity, MUC1 molecules enter the bloodstream. Circulating MUC1 levels are measured by CA15-3 assay. Clinical utility of CA15-3 is in monitoring the response to therapy, in detecting early disease recurrence, and in assessing prognosis of patients with breast cancer [80–83]. Elevated preoperative levels of CA15-3 are associated with poor prognosis. Moreover, even in the absence of measurable disease, increasing levels of CA15-3 indicate a treatment failure [74, 83]. Clinical utility of this marker in other cancers is still under investigation.

CA19-9 assay detects aberrant sialyl Lewis^a^ glycan (SLe^a^) that is expressed on glycolipids and glycoprotein in patients with gastrointestinal malignancies. This marker is useful to monitor clinical response to therapy and to evaluate the prognosis of patients with pancreatic, colorectal, gastric, or biliary cancer. CA19-9 is neither tumour specific nor organ specific but has the highest sensitivity and specificity for pancreatic cancer. Levels of this marker strictly correlate with the clinical response to treatment and can predict the recurrence of tumour after pancreatectomy prior to clinical or instrumental evidence of disease [23, 24]. In colon cancer, patients with higher levels of CA19-9 have in the first three years after diagnosis a mortality rate four times higher than those with lower levels. In gastric cancer, CA19-9 levels before surgery are a prognostic marker and an independent risk factor for the relapse of gastric carcinoma [25–27, 84].

CA72-4 assay is used to detect aberrant sialyl-Tn (STn), a carbohydrate antigen expressed in mucins. Elevated levels of this antigen are detected in serum of patients with various epithelial cancers, such as gastric, colorectal, pancreatic, lung, and breast carcinomas, and are usually associated with poor prognosis, tumour metastasis, and a decreased survival [15–17, 21, 22, 26, 48, 85, 86]. CA72-4 assay is used for monitoring gastric carcinoma, given that a relevant serological level increase is considered a predictor of tumour recurrence [26]. Moreover, CA72-4 is an independent prognostic factor in gastric carcinoma and in pancreatic cancer [87, 88].

An additional serological assay measures the serum levels of carcinoembryonic antigen (CEA), a member of a family of N-glycoproteins, that is overexpressed in a wide range of human carcinomas, including colorectal, gastric, pancreatic, lung, and breast carcinomas, and is shed into circulation [25, 27, 80, 82, 83, 89–93]. However, moderate to significant increase in serum CEA levels is observed in a variety of chronic and acute inflammatory diseases that are unrelated to cancer, including alcoholic cirrhosis, cholelithiasis, obstructive jaundice, cholangitis, liver abscess, emphysema, bronchitis, gastric ulcer, gastritis, diverticulitis, diabetes, and collagen vascular diseases [88, 94]. Thus, even though the serum CEA level is measured in patients with colorectal cancer for prognosis and clinical follow-up of patients [95, 96], it cannot be used for screening because of its low sensitivity in early stages of colorectal cancer.

Improved specificity and diagnostic capability can be achieved by developing assays that detect cancer-specific alterations of glycoprotein glycosylation. This is the case of alpha-fetoprotein (AFP), a marker of HCC. AFP is not HCC specific because elevated serum levels of AFP are also found in other conditions such as pregnancy, hepatitis, and liver cirrhosis. Total AFP can be separated into three glycoforms; of those the AFP-L3 glycoform, consisting of core-fucosylated AFP, has been shown to be highly specific for HCC even at early stage of disease and useful for early tumor recognition [37].

## 4. CD43 Is a Mucin-Type Cancer-Associated Glycoprotein

CD43, also named leukosialin, sialophorin, galactoglycoprotein, leukocyte sialoglycoprotein, and is a mucin-like type I transmembrane protein. In humans, it is expressed in haematopoietic cells, including T lymphocytes, monocytes, granulocytes, natural killer cells, platelets, and haematopoietic stem cells, with the exception of mature erythrocytes and B cell subpopulations [97–103].

The human CD43 protein is encoded by a single gene on chromosome 16 (gene map locus 16p11.2) and comprises the signal peptide of 19 amino acids at the amino terminus followed by a highly glycosylated extracellular region of 235 amino acids, the transmembrane region of 23 amino acids, and the intracellular carboxy-terminal region of 123 amino acids [104, 105]. The mucin-like extracellular domain has an extended rod-like structure that protrudes about 45 nm from the cell surface and is serine and threonine rich, thus enabling an extensive *O*-glycosylation (about 80 *O*-linked glycan chains). Only one potential N-glycosylation site is located near to the transmembrane domain at position N239 [106, 107]. The intracellular region contains a number of potential phosphorylation sites that can mediate transduction of activation signals. The unglycosylated form of CD43 migrates on SDS-PAGE with a molecular weight of 54 kDa [97], whereas CD43 from different haematopoietic cell lines displays increased molecular weights since it is glycosylated with *O*-linked chains that differ in core structure and sialylation [101, 108, 109]. Two CD43 glycoforms have been described in haematopoietic cells depending on cell type and cellular activation. Resting T lymphocytes express mostly leukosialin with an apparent molecular weight of 115 kDa, which contains almost exclusively tetrasaccharides, while activated T cells, monocytes, neutrophils, and platelets express a glycoform with the apparent molecular weight of 130 kDa, which possesses mainly branched hexasaccharides [97, 98, 110]. CD43 glycoforms can be coexpressed on the surface of the same cell [111] suggesting that they are functionally distinct. CD43 has a negative net charge due to the high content of sialic acid in the *O*-glycan chains [104]. In particular, CD43 can express carbohydrate structures, such as the sialyl-Lewis^x^ (SLe^x^) epitope, a ligand for P- and E-selectin, which favour cell-cell interactions [112]. Simultaneous expression of different CD43 glycoforms on the cell surface suggests that they can finely regulate cell-cell interactions [111, 113, 114]. The CD43 extracellular domain can be cleaved by metalloprotease and serine protease from the cell surface of stimulated granulocytes and lymphocytes, and the soluble extracellular fragment can be detected in normal human sera [115–118]. The cytoplasmic domain is evolutionarily conserved; it is involved in signal transduction mediating the connection to the cytoskeleton through binding to ezrin, radixin and moesin (ERM) proteins and contains a nuclear localization signal (NLS), which explains the nuclear localization of CD43, and a proline-rich sequence resembling SH3 binding consensus [119–123].

CD43 expression on human embryonic stem cell (hESC)-derived haematopoietic progenitors may suggest a role of CD43 in haematopoietic development [124]. Further, CD43 is involved in the regulation of cell activation, differentiation, adhesion, and migration and may play a role in immune response by modulating cell growth, survival, and apoptosis [113, 114, 125]. However, some functions attributed to CD43 seem to be contradictory. In fact, CD43 was found to play a proadhesive or an antiadhesive role, and to induce apoptosis or to protect cells against apoptosis [113, 114]. Indeed, CD43 induced cellular adhesion through the binding to molecules such as E-selectin [126, 127], galectin-1 and galectin-3 [128], siglec-1 [129], M-ficolin [130], integrins [131], cell surface nucleolin [132], and ICAM-1 (intercellular adhesion molecule type 1) [133]. However, it was reported that the antiadhesive property of CD43 depends not only on the strong negative charge and the large extended structure of the extracellular region, but also on interaction of the cytoplasmic domain with the cytoskeleton [134]. Most of the studies concerning the CD43 signalling have been performed in haematopoietic cells as this glycoprotein has been considered for a long time an exclusive marker of leukocytes. Indeed, the CD43 intracytoplasmic tail is involved in cell signalling and regulation of surface expression of CD43 during processes such as cell migration and immunological synapse. In particular, the CD43 intracytoplasmic domain binds to the ERM adaptor proteins, which cross-link actin filaments to transmembrane proteins such as CD43, CD44, and ICAM-2 [121], and interacts with Src kinases and zeta-chain of CD3 upon CD43 activation [122].

A growing number of reports indicate that there is an association between CD43 and cancer. One reason for CD43 involvement in cancer development is that CD43 signalling induces the activation of *β*-catenin, NF-*κ*B [135, 136], NFAT, and AP-1, which are prosurvival transcription factors that can promote tumorigenesis when deregulated [137, 138]. The first study concerning the expression of CD43 in tumour cells of nonhaematopoietic origin reported that CD43 was found in the colon carcinoma cell line COLO 205. Indeed, an aberrant CD43 glycoform with the apparent molecular weight of 200 kDa was described in COLO 205 [139, 140]. Subsequently, CD43 glycoforms were detected in a variety of cancer cell lines [139, 141, 142] and in different tumours of nonhaematopoietic origin, including lung, breast, and colon, and not in the normal counterparts [143–147]. Further, alterations of the CD43 glycosylation pattern were associated with severe immunodeficiency [148, 149]. These findings indicate that an aberrant expression of CD43 glycoforms may have a role in cancer development [144, 145, 150, 151]. Indeed, in many types of cancer, *O*-glycans show heterogeneity and altered levels and may generate unusual epitopes. For example, they can be highly sialylated, less sulphated, or truncated and contain the T and Tn antigens and their sialylated forms. These structural changes may have many biological and pathological effects because they influence the ligand-receptor binding that mediates the interactions of cancer cells with their microenvironment. Hence, these events interfere with the proliferation of the cells, their ability to invade and metastasise, and their interactions with lectins, adhesion molecules, and other cell surface receptors of immune cells [31, 42]. For example, increased sialylation of *O*-glycans has been shown to be associated with the enhanced growth rate of breast cancer cells in transgenic mice [38], with sialyl-Tn being a poor prognosis marker for cancer patients. Increased sialylation has also been associated with metastatic potential of cancer cells [39]. Sialyl-Lewis structures that are present in CD43 are often overexpressed by cancer cells; in colon cancer patients, metastatic tumour cells expressed increased amounts of sialyl-Lewis^a^, sialyl-Lewis^x^, and sialyl-dimeric Lewis^x^ as compared with primary tumour cells [28–30, 152, 153]. These sialyl-Lewis structures are ligands for selectins that are involved in the attachment of leukocytes to the endothelium, suggesting that cancer cells may use the sialyl-Lewis^x^-selectin-binding mechanism during tumour invasion and metastasis [154]. In fact, it has been found that high metastatic tumour cells expressing higher levels of sialyl-Lewis antigens adhere more strongly to E-selectin expressing cells compared to their low metastatic counterparts [155, 156]. In addition, CD43 is involved in tumour cell adhesion and in development of metastasis via interaction with its ligand ICAM-1 [142].

A feature of CD43 expression in nonhaematopoietic cancer cells is that it has an intracellular localization in contrast to cell membrane expression characteristic of leukocytes [146, 150]. Like in leukocytes, the CD43 extracellular domain in cancer cells is cleaved and stored inside the cell before exocytosis [140]. Moreover, processing of CD43 by *γ*-secretase results in a CD43 cytoplasmic tail fragment, containing a functional nuclear localization sequence interacting with the nuclear transporter protein Ran. CD43 cytoplasmic tail has been found to translocate to the nucleus and is involved in the regulation of apoptosis, given that inhibition of either its nuclear translocation or its release by gamma-secretase was found to be proapoptotic [137, 157, 158]. Consequently, abnormal glycosylation could modify the proteolytic processing of CD43 and, therefore, interfere with CD43 functionality.

## 5. UN1 Monoclonal Antibody Recognizes Cancer-Associated CD43 Glycoforms

Peculiar glycoforms of CD43 are recognized by the UN1 monoclonal antibody (mAb), which was originally produced in our laboratory [159]. The UN1 mAb was selected for a high reactivity against human immature thymocytes (CD3dim) [159] and later shown to recognize an epitope of the UN1/CD43 antigenic glycoprotein, which includes a GalNAc-*O*-linked monosaccharide [147, 160, 161]. The UN1/CD43 antigen was shown to be expressed in some leukemic T-cell lines, such as HPB-ALL, H9, MOLT-4, and in human thymocytes and a subpopulation of peripheral blood CD4+ T-lymphocytes and not in other blood cells [159, 160, 162, 163]. Moreover, the UN1/CD43 antigen was expressed at early stages of development in fetal tissues, including thymus, spleen, adrenal cortex, bronchial epithelium, and skin, and was downregulated during ontogenesis [164]. The involvement of UN1/CD43 glycoforms in oncogenesis was suggested by several findings. In fact, UN1/CD43 was detected in a variety of solid tumours, including breast, colon, gastric, and squamous cell lung carcinomas, while it was undetected in the corresponding normal tissues and benign lesions [147, 164, 165]. In particular, the expression level of UN1/CD43 glycoforms in breast cancer cells increased with the progression stage of the disease [165]. In fact, UN1 was not expressed in normal cells and nonproliferative lesions, while it was poorly expressed in fibroadenoma, moderately expressed in atypical hyperplasia and *in situ* breast carcinoma (stage 0 of disease), and highly expressed in infiltrating breast carcinoma (stages I–III) with the highest expression level in metastatic lesions (stage IV) [165]. These results underscore a direct correlation between its expression and breast cancer progression. Due to the wide expression in fetal tissues and down-regulation during ontogeny with reexpression in cancer cells, the UN1/CD43 glycoforms were considered an oncofetal antigen [164]. In this regard, UN1 represents an interesting marker of potential value for immunophenotyping studies and clinical applications in cancer diseases [164, 165], besides the usefulness for studies on the role of CD43 glycosylation in tumorigenesis [147].

## 6. Conclusions

It has been well known for a long time that glycosylation is a very significant posttranslational modification of many biologically important molecules and that aberrant glycosylation of glycan structures is a common feature of neoplastic transformation. Many clinical cancer biomarkers correspond to glycosylated molecules and the alterations in their glycan moieties can be utilized as a target to improve existing cancer biomarkers. Glycomics and glycoproteomics are needed for the discovery of new glycan biomarkers with better sensitivity and specificity for early detection of cancer, for evaluation of therapeutic efficacy of cancer treatment, and for assessment of prognosis. CD43 is a mucin-like sialoglycoprotein, considered for a long time an exclusive marker of leukocytes but subsequently, found to be expressed in cancers, showing altered glycosylations. The UN1 mAb identifying cancer-associated CD43 glycoforms may represent an interesting tool for diagnostic and therapeutic purposes.

## Figures and Tables

**Table 1:** Cancer-associated glycan alterations observed in the referred studies.

Structure	Findings	References
T antigen	Overexpression	[14–16]
Sialyl-T antigen	Overexpression	[12, 14]
Tn antigen	Overexpression	[17–20]
Sialyl-Tn antigen	Overexpression	[17, 18, 20–22]
Sialyl-Lewis^a^	Overexpression	[23–27]
Sialyl-Lewis^x^	Overexpression	[28–30]
Core-2	Overexpression/underexpression	[12, 31]
Core-3	Underexpression	[12, 31]
Core-4	Underexpression	[12, 31]
*β*1-6 branching of N-glycans	Overexpression	[32, 33]
Fucosylation	Overexpression	[34–37]
Sialylation	Overexpression	[38, 39]
